# Hypermethylation of CCND2 in Lung and Breast Cancer Is a Potential Biomarker and Drug Target

**DOI:** 10.3390/ijms19103096

**Published:** 2018-10-10

**Authors:** Chin-Sheng Hung, Sheng-Chao Wang, Yi-Ting Yen, Tzong-Huei Lee, Wu-Che Wen, Ruo-Kai Lin

**Affiliations:** 1 Division of Breast Surgery, Department of Surgery, Taipei Medical University Hospital, Taipei 110, Taiwan; hungcs@tmu.edu.tw; 2 Department of Surgery, School of Medicine, College of Medicine, Taipei Medical University, Taipei 110, Taiwan; 3 Division of General Surgery, Department of Surgery, Shuang Ho Hospital, Taipei Medical University, New Taipei City 235, Taiwan; 4 Ph.D Program in Biotechnology Research and Development, College of Pharmacy, Taipei Medical University, Taipei 110, Taiwan; wsc0327@gmail.com; 5 Professional Master Program in Pharmaceutics and Biotechnology, College of Pharmacy, Taipei Medical University, Taipei 110, Taiwan; chocayen0822@gmail.com; 6 Institute of Fisheries Science, National Taiwan University, Taipei 110, Taiwan; thlee1@ntu.edu.tw; 7 Golden Biotechnology Corporation, 15F., No. 27-6, Sec. 2, Zhongzheng E. Rd, Taipei, TW 110, Taiwan; wwc@goldenbiotech.com.tw; 8 Graduate Institute of Pharmacognosy, Ph.D. Program for the Clinical Drug Discovery from Botanical Herbs; Master Program for Clinical Pharmacogenomics and Pharmacoproteomics, Taipei Medical University, Taipei 110, Taiwan

**Keywords:** lung adenocarcinoma, triple-negative breast cancer (TNBC), hypermethylation, *Antrodia camphorata*, antroquinonol D, *CCND2*, tumor suppressor gene, prognostic factor, circulating cell-free DNA

## Abstract

Lung and breast cancer are the leading causes of mortality in women worldwide. The discovery of molecular alterations that underlie these two cancers and corresponding drugs has contributed to precision medicine. We found that CCND2 is a common target in lung and breast cancer. Hypermethylation of the *CCND2* gene was reported previously; however, no comprehensive study has investigated the clinical significance of *CCND2* alterations and its applications and drug discovery. Genome-wide methylation and quantitative methylation-specific real-time polymerase chain reaction (PCR) showed *CCND2* promoter hypermethylation in Taiwanese breast cancer patients. As compared with paired normal tissues and healthy individuals, *CCND2* promoter hypermethylation was detected in 40.9% of breast tumors and 44.4% of plasma circulating cell-free DNA of patients. The western cohort of The Cancer Genome Atlas also demonstrated *CCND2* promoter hypermethylation in female lung cancer, lung adenocarcinoma, and breast cancer patients and that *CCND2* promoter hypermethylation is an independent poor prognostic factor. The cell model assay indicated that CCND2 expression inhibited cancer cell growth and migration ability. The demethylating agent antroquinonol D upregulated CCND2 expression, caused cell cycle arrest, and inhibited cancer cell growth and migration ability. In conclusion, hypermethylation of *CCND2* is a potential diagnostic, prognostic marker and drug target, and it is induced by antroquinonol D.

## 1. Introduction

Lung cancer and breast cancer are the most common and leading causes of mortality in women worldwide [1,2]. The discovery of various molecular, genetic and epigenetic alterations that underlie lung cancer and breast cancer has improved the understanding of their tumorigenesis and it has contributed to the development of specifically targeted therapies employing protein overexpression inhibitors and specific mutation inhibitors, such as epidermal growth factor receptor (EGFR) and human epidermal growth factor receptor 2 (HER2) [3,4,5].

In lung cancer, adenocarcinomas have been historically the most frequent subtype, and the prevalence rate of lung adenocarcinoma continues to increase among females worldwide [6]. Lung adenocarcinoma is often diagnosed late when local invasion is considerable or metastasis has already appeared. Therefore, the mortality at five years remains very high, ranging from 51% to 99%, depending on the stage [7]. In breast cancer, triple-negative breast cancer (TNBC) is a complicated and aggressive subtype of breast cancer that lacks estrogen receptors, progesterone receptors, and HER2 amplification, making it difficult to target therapeutically. TNBC has the highest rate of metastatic disease and the poorest overall survival of all breast cancer subtypes [8]. Therefore, a novel diagnostic biomarker and candidate treatment target specific for lung adenocarcinoma and TNBC was investigated in this study. We analyzed data from The Cancer Genome Atlas (TCGA). TCGA has profiled more than 10,000 samples that were derived from 33 types of cancer to improve our understanding of the molecular bases of cancer and advancing our ability to treat cancer with novel therapeutic methods [9]. We found that *CCND2* was significantly promoter hypermethylated and lower when expressed in lung adenocarcinoma and TNBC as compared with adjacent normal tissues and other cancers, such as colon cancer, liver cancer, gastric cancer, and esophageal cancer. *CCND2* encodes cyclin D2, a protein involved in cell cycle progression that is thought to act as a regulator of cyclin-dependent kinases 4 and 6 (CDK) 4/6 in the G1–S transition [10]. The CDK4/6 inhibitor of CDK4 (INK4)-retinoblastoma (Rb) pathway plays a crucial role in cell cycle progression, and its dysregulation is an important contributor to endocrine therapy resistance in breast cancer [11]. However, hypermethylation of the *CCND2* gene is common in many cancers, including lung and breast cancer, and it results in low *CCND2* expression [12,13,14,15]. *CCND2* hypermethylation is significantly more frequent in invasive peripheral lung adenocarcinoma [16]. *CCND2* hypermethylation, and, therefore, low *CCND2* protein expression are associated with a poor prognosis in epithelial ovarian cell cancer [17] and hepatocellular carcinoma recurrence [18]. However, no comprehensive study has investigated the clinical significance of *CCND2* alterations and its applications and drug discovery. In this study, we found that low *CCND2* expression was associated with a poor prognosis, especially in patients with non-small cell lung cancer (NSCLC) or breast cancer; thus, *CCND2* might serve as a potential drug target. Therefore, we investigated whether *CCND2* is a potential drug target and it is identified a *CCND2* inducer drug.

We previously discovered a fungal-derived Taiwanese natural product, antroquinonol D (3-demethoxyl antroquinonol), which is an analog of antroquinonol. Antroquinonol D can insert into the putative cytosine pocket and compete with the cofactor SAM, resulting in decreased DNMT1 activity [19]. Antroquinonol and antroquinonol D are isolated from *Antrodia camphorata*, the wild fruiting body of which has been used by aboriginal peoples in Taiwan to treat hepatitis, cirrhosis, liver cancer, diarrhea, abdominal pain, hypertension, and itching [20]. Antroquinonol inhibits the migration ability and proliferation in lung, breast, liver, and pancreatic cancer cells via ERK-AP-1, AKT-NF-kappaB, and PI3K/mTOR pathways [19,21,22,23,24,25]. Antroquinonol D has anticancer potential through the suppression of DNA methyltransferase enzyme activity and demethylation and the reactivation of tumor-suppressing genes in breast cancer [19]. Antroquinonol is currently in phase II trials (USA and Taiwan) for NSCLC and it was recently granted orphan drug status by the U S Food and Drug Administration for pancreatic cancer and acute myeloid leukemia [26,27]. The total synthesis of (+)-antroquinonol and (+)-antroquinonol D, two structurally unique quinonols with a sesquiterpene, has been reported [28,29].

In this study, we further investigated whether *CCND2* is a potential drug target and identified a *CCND2* inducer drug.

## 2. Results

### 2.1. CCND2 is a Common Target in Lung and Breast Cancer

**Figure 1:**
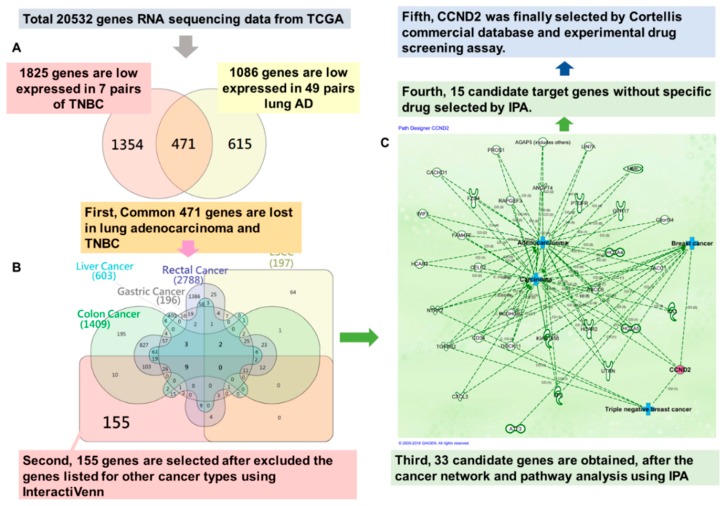
*CCND2* is a common drug target for lung adenocarcinoma and triple-negative breast cancer. (**A**) The Venn diagram showed the number of intersections of lung adenocarcinoma (lung AD) and triple-negative breast cancer (TNBC). (**B**) The Venn diagram displayed the intersections among 7 types of cancer. (**C**) Thirty-three genes were involved in lung adenocarcinoma and triple-negative breast cancer, as analyzed using Ingenuity Pathway Analysis software. The red circle represents CCND2 protein. The blue cross represents the related tumor types.

To identify a novel treatment target that is specific to lung adenocarcinoma and triple-negative breast cancer (TNBC), we analyzed the RNA sequencing data from TCGA and identified a candidate common target using InteractiVenn and Ingenuity Pathway Analysis. We analyzed 49 pairs of lung adenocarcinoma, seven pairs of TNBC, 13 pairs of esophageal cancer, 32 pairs of gastric cancer, 50 pairs of liver cancer, six pairs rectal cancer, and 41 pairs of colon cancer. We included genes from the intersection list of aberrant low expression for lung adenocarcinoma and TNBC (Figure 1A), but excluded the genes that were listed for other cancer types using InteractiVenn (Figure 1B). After network and pathway analysis using Ingenuity Pathway Analysis, we obtained 33 candidate target genes (Figure 1C). Finally, 15 candidate target genes without drug or specific inhibitors in previous reports were further selected by Ingenuity Pathway Analysis (Table S1). In our previous study, we observed that antroquinonol D could decrease methylation and induce *CCND2* expression in breast cancer MDA-MB-231 cells (Table S2). Therefore, *CCND2* was finally selected for further analysis.

### 2.2. CCND2 Reveals Promoter Hypermethylation and mRNA Downregulation in Lung Cancer and Breast Cancer

**Figure 2:**
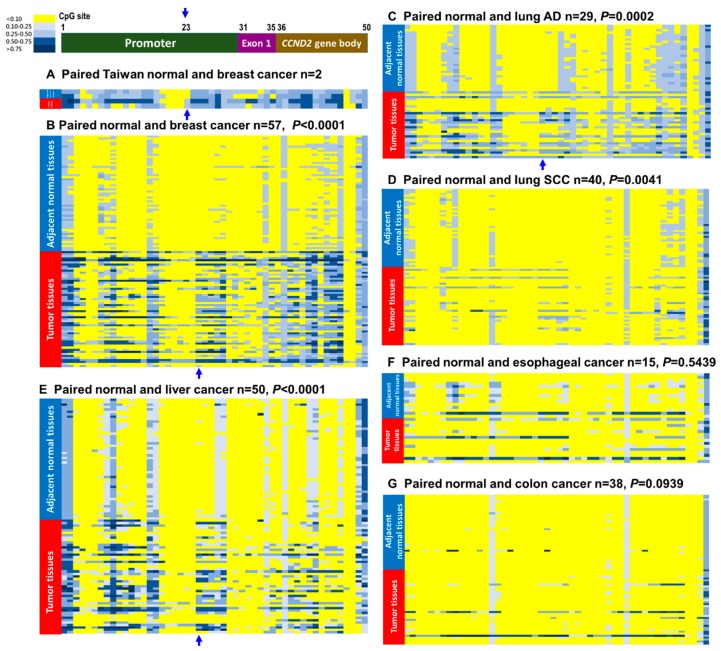
*CCND2* methylation pattern in breast, lung, liver, esophageal, and gastric cancers. The 50 CpG sites of the *CCND2* methylation levels from the promoter region (30 CpG sites), exon 1 region (5 CpG sites), and gene body region (15 CpG sites, sites 36–46 on intron 1, sites 47–49 on intron 2, site 50 on intron 4) were identified using the Illumina Methylation 450K array-based assay. The *CCND2* gene is located on chromosome 12. Positions of CpG from 1 to 50 are located from 4381435 to 4390091 of chromosomes 12. (**A**) Paired breast tumors and adjacent normal tissues from breast cancer patients. The red arrow indicates the differential site between breast normal and cancerous tissues. (**B**) The data for methylation patterns were collected from The Cancer Genome Atlas (TCGA). Paired cancer and adjacent normal tissues were obtained from breast cancer (*n* = 57), (**C**) lung adenocarcinoma (*n* = 29), (**D**) lung SCC (*n* = 40), (**E**) liver cancer (*n* = 50), (**F**) esophageal cancer (*n* = 15), and (**G**) colon cancer (*n* = 38). The blue arrow indicates the differential sites between breast normal and cancerous tissues. Adjacent normal tissues from patients are marked with blue bars, and tumor tissues are marked with red bars.

Reduced *CCND2* expression has been reported in various cancers, and the mechanism underlying *CCND2* silencing in these cases was aberrant promoter methylation [14,15,16,30]. To verify whether *CCND2* showed promoter hypermethylation in Taiwanese breast cancer patients, we performed qMSP to analyze the methylation levels in 93 tumors and paired adjacent normal tissues of breast cancer patients. We found 38 of 93 (40.9%) of breast cancer patients showed *CCND2* promoter hypermethylation. In addition, *CCND2* promoter hypermethylation was detected in plasma circulating cell-free DNA from 8 of 18 (44.4%) of breast cancer patients, and little *CCND2* promoter hypermethylation was detected in women without breast cancer (3 of 19, 15.8%). We compared the methylation status between patients from Taiwan and Western countries while using the human Methylation 450K BeadChip array, which showed that Taiwanese breast cancer patients had generally higher methylation levels in the *CCND2* promoter and exon 1 regions than patients from western countries in the TCGA dataset. An increase in the methylation level of the *CCND2* promoter CpG site cg22678952 when compared with paired adjacent normal tissues was found in Taiwanese breast cancer patients (Figure 2A). Furthermore, the *CCND2* promoter and exon 1 regions exhibited significantly increased methylation levels in breast tumors compared with paired adjacent normal tissues of breast cancer in TCGA (Figure 2B). Notably, hypermethylation of *CCND2* was associated with female lung cancer and lung adenocarcinoma (Table S3, *p* < 0.001). Further analysis of the methylation patterns of other cancer types revealed that the *CCND2* promoter hypermethylation was significant in breast cancer, lung adenocarcinoma, and liver cancer (Figure 2C,E, Table 1 and Table S4), but not in esophageal and colon cancers (Figure 2F,G and Table 1).

**Figure 3:**
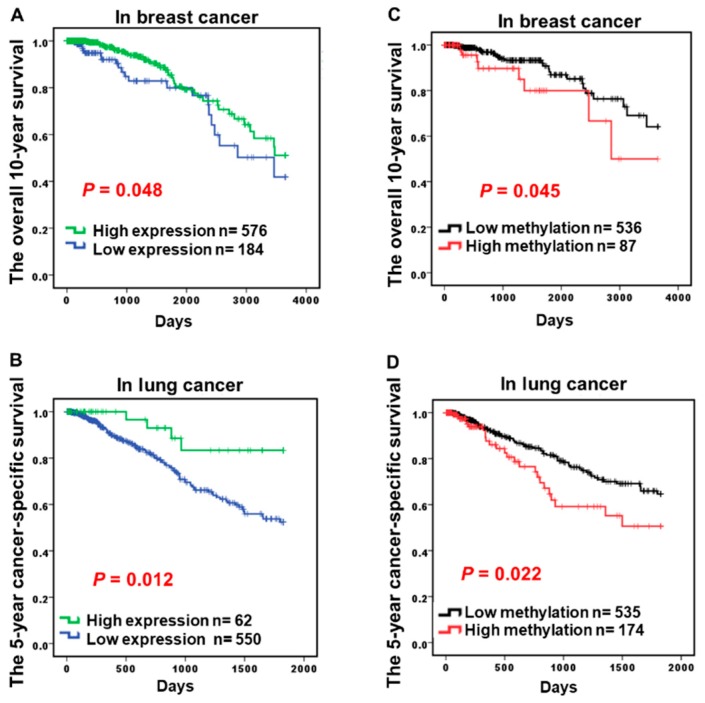
*CCND2* promoter hypermethylation and mRNA expression level and its correlation with survival in lung, breast, and liver cancer patients Data for CCND2 DNA hypermethylation and the mRNA expression level assayed by the Illumina Methylation 450K array-based assay and RNA sequencing and survival data were collected from TCGA. (**A**,**B**) Kaplan-Meier survival curves were used to compare the survival between cancer patients with low and high *CCND2* mRNA expression levels in breast cancer and lung cancer patients. (**C**,**D**) Kaplan–Meier survival curves were used to compare the survival between cancer patients with low and high *CCND2* methylation levels in breast cancer and lung cancer patients.

Regarding *CCND2* mRNA expression, 91.8% (45/49) of lung adenocarcinoma patients, 84.9% (62/73) of breast cancer patients, and 100% (7/7) of TNBC patients showed *CCND2* mRNA downregulation in tumors when compared with that in adjacent normal tissues (Table 1, Figure 3A, all *p* < 0.001), but the difference was not significant in esophageal, gastric, liver, and colon cancers (Table 1 and Figure 3A). In addition, 63.3% of tumors from breast patients with methylated *CCND2* were also characterized by low *CCND2* mRNA expression (Table 1).

### 2.3. Hypermethylation of CCND2 is an Independent Poor Prognostic Factor

To determine whether *CCND2* is involved in the tumor progression and prognosis of lung cancer and breast cancer, *CCND2* mRNA expression and clinical parameters of lung and breast cancer were analyzed in 760 breast cancer patients and 612 lung cancer patients from the TCGA datasets. Low *CCND2* expression was associated with a poor survival in breast cancer and lung cancer patients while using the log-rank test (Figure 3A,B, *p* = 0.048 and *p* = 0.012, respectively), but this association was not found in colon cancer, esophageal cancer, and gastric cancer patients (Table 1, Figure S1). Aberrant methylation in the *CCND2* promoter was also associated with a poor prognosis in breast cancer and lung cancer using the log-rank test (Figure 3C,D, *p* = 0.045 and *p* = 0.022, respectively). Adjusted for sex, race, age, tumor type, and stage, the multivariate Cox proportional hazard model analysis showed a significant correlation of *CCND2* expression with the five-year overall survival in lung cancer and 10-year overall survival in breast cancer (Table 2, *p* = 0.021 and 0.001, respectively). Aberrant methylation in the *CCND2* promoter was also associated with a poor prognosis in lung cancer and breast cancer using multivariate Cox proportional hazard model analysis (Table 2, *p* = 0.039 and *p* = 0.009). Therefore, *CCND2* is an independent prognostic factor. In conclusion, alterations of CCND2 serve as a therapeutic target for precision medicine in lung cancer and breast cancer.

### 2.4. CCND2 is Involved in the Inhibition of Lung Cancer Cell Growth and Cell Migration

**Figure 4:**
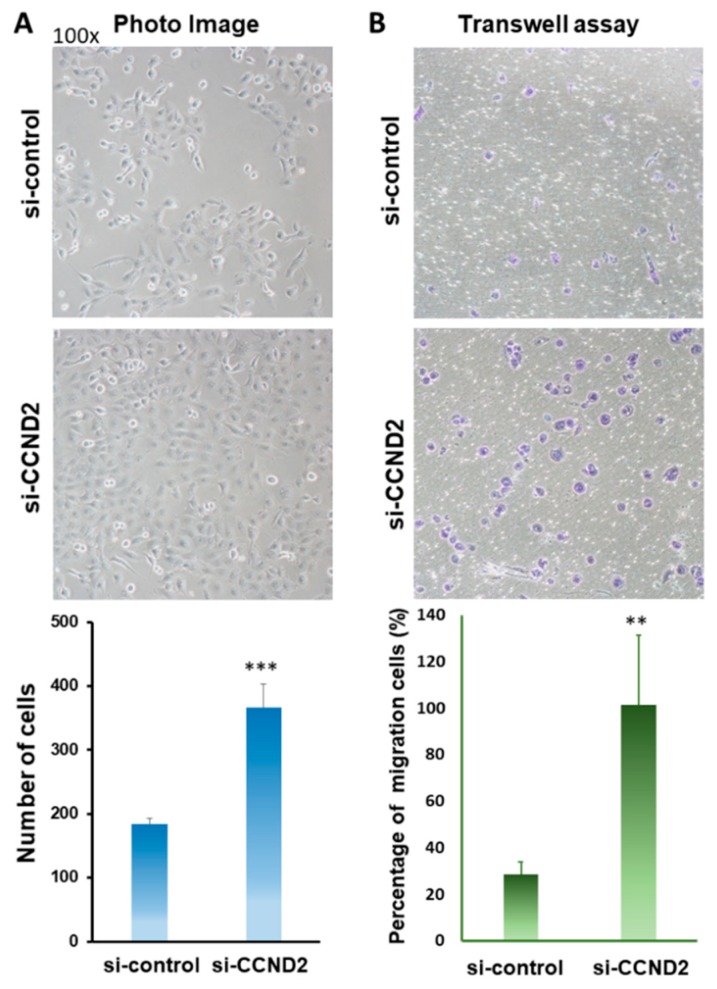
*CCND2* controls cell growth and motility (**A**) Photograph of *CCND2* knockdown-induced H1299 lung cancer cell proliferation (upper panel, original magnification 100×) and bar graph demonstrating the relative cell proliferation rates assayed by cell counting (lower panel). (**B**) The Transwell assay was used to further investigate the migration abilities of H1299 cancer cells. The images represent treatment with si-control and si-*CCND2* (upper panel, original magnification, 100×). The average number of migration cells (those that passed through the membrane) was counted in five different fields (lower panel). ** *p* = 0.006, *** *p* ≤ 0.001.

Clinical data have suggested that *CCND2* was down-regulated in lung cancer and it was associated with lymph node metastasis, distant metastasis, and a poor prognosis (Table S3, *p* = 0.014, and *p* = 0.017, respectively). To determine whether *CCND2* expression is underexpressed in lung cancer cells, A549, CL1-5, H1299, and normal lung IMR-90 were analyzed through real-time RT–PCR and Western blotting. The results indicated that *CCND2* was expressed in normal lung cells, but it was underexpressed in lung cancer cells (Figure S2). To investigate whether *CCND2* is associated with lung cancer cell migration, knockdown by si-*CCND2* was performed in H1299 lung cancer cells for 72 h (Figure S3). Furthermore, cell motility was analyzed using Transwell assays. The data revealed that the knockdown of *CCND2* increased the growth rate and migration ability of H1299 cells by 2.09-fold and 3.53-fold, respectively (Figure 4B).

### 2.5. The Candidate CCND2 Stimulator Antroquinonol D Induces CCND2 mRNA and Protein Expression

**Figure 5:**
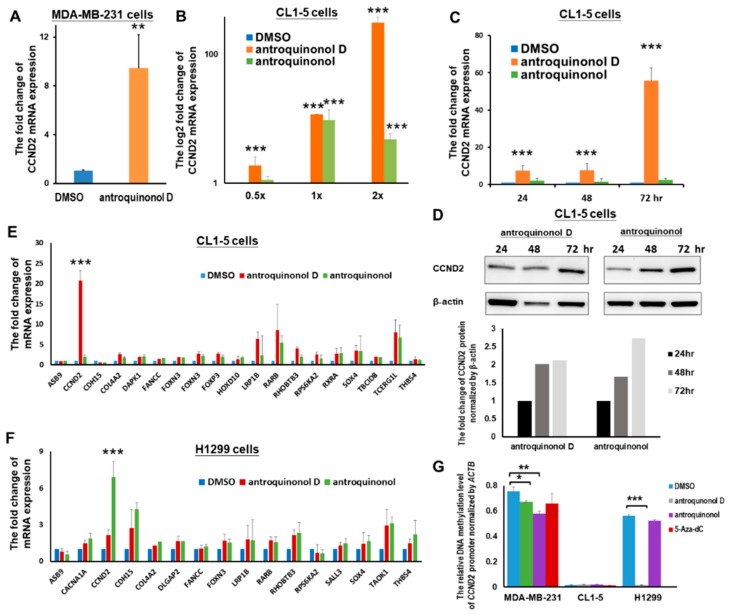
*CCND2* can be induced by antroquinonol D and antroquinonol. CCND2 mRNA expression was induced by antroquinonol D in the MDA-MB-231 breast cancer cell line (**A**) and in lung adenocarcinoma cell line CL1-5 in a dose-dependent manner (**B**). The *CCND2* mRNA (**C**) and protein expression levels (**D**) were also induced in a time-dependent manner. (**E**) Antroquinonol D induced *CCND2* mRNA expression compared with that of other tumor suppressor genes in CL1-5. The antroquinonol D analog antroquinonol also induced *CCND2* expression in the H1299 lung cancer cell line (**F**). (**G**) CCND2 DNA methylation levels were analyzed by qMSP. The data are presented as the means ± SD. * *p* ≤ 0.05, ** *p* ≤ 0.01, *** *p* ≤ 0.001.

*CCND2* inhibits lung cancer migration and it was found to be a good prognostic factor; thus, it can serve as an ideal drug target. However, no *CCND2* inducer has been found. Candidate CCND2 stimulators were surveyed using IPA^®^ (Ingenuity^®^ Pathway Analysis; Qiagen) and Cortellis (Cortellis; Clarivate Analytics). However, no result was found for CCND2 inhibitors or stimulators. In our previous study, antroquinonol D inhibited MDA-MB-231 breast cancer cell growth and induced DNA demethylation, likely through the inhibition of DNA methyltransferase 1 activity [19]. In the present study, we observed that antroquinonol D induced *CCND2* expression in breast cancer MDA-MB-231 cells (Figure 5A), and both antroquinonol D and antroquinonol could induce *CCND2* expression in lung cancer CL1-5 cells in a dose-dependent and time-dependent manner (Figure 5B,C). *CCND2* expression was increased by antroquinonol D in CL1-5 cells and by antroquinonol in H1299 cells. *CCND2* mRNA expression was increased by 11.57-fold and 309.37-fold with 15 μM (IC50) and 30 μM antroquinonol D, respectively, at 72 h and by 7.29-fold, 7.50-fold, and 55.66-fold at 24, 48, and 72 h, respectively (Figure 5B,C). The *CCND2* protein was also detected at a 2.1-fold and 2.7-fold increase following antroquinonol D and antroquinonol treatment for three days (Figure 5D). To analyze whether antroquinonol D and antroquinonol specifically induced *CCND2* expression, real-time RT–PCR of multiple tumor suppressor gene levels was performed in lung cancer cells CL1-5 and H1299. *CCND2* was specifically induced by 20.69-fold and 2.17-fold following 15 μM antroquinonol D treatment for three days in CL1-5 and H1299 cells, respectively (Figure 5E,F). *CCND2* mRNA expression was increased by 2.03-fold and 6.93-fold with antroquinonol in CL1-5 and H1299 cells, respectively (Figure 5E,F).

To investigate whether antroquinonol D induced *CCND2* expression through DNA demethylation, we analyzed changes in genomic methylation using the Illumina Methylation 450K array-based and pyrosequencing assay. Treatment with 15 μM antroquinonol D for three days decreased the methylation status of several genes in CL1-5 lung cancer cells. However, there was no change in the DNA methylation level for *CCND2* (Figure S4). We designed the primers targeting the promoter and exon 1 regions of *CCND2* and detected the DNA methylation level through pyrosequencing and qMSP. The CpG island methylation levels in the *CCND2* genes displayed a decrease in promoter region and few changes in exon 1 region following treatment with antroquinonol D (Figure 5G and Table S5). Therefore, we suggest that antroquinonol D induced *CCND2* expression, but it was mediated in both DNA demethylation-independent and dependent manner.

### 2.6. Antroquinonol and Antroquinonol D Inhibit Growth and Induce Cell Cycle Arrest in the G1 Phase in a Dose-Dependent Manner in Lung Cancer Cell Lines

In our previous study, antroquinonol D inhibited breast cancer cell proliferation and migration without any damage to normal breast cells [19]. To determine whether it could inhibit the cell proliferation of CL1-5, H1299, and A549 lung cancer cells, we treated the normal cell line IMR-90 and three lung cancer cell lines—CL1-5, H1299, and A549—for 72 h. Antroquinonol D suppressed the growth of all cell lines at three days (Figure S5). Antroquinonol led to 50% cell death in CL1-5 at 1.25 µM and 40% cell death in IMR-90 at a high dose of 20 µM (Figure S5). However, antroquinonol D exerted minor toxicity to IMR-90 but 40–80% lung cancer cell death at 20 µM (Figure S5).

**Figure 6:**
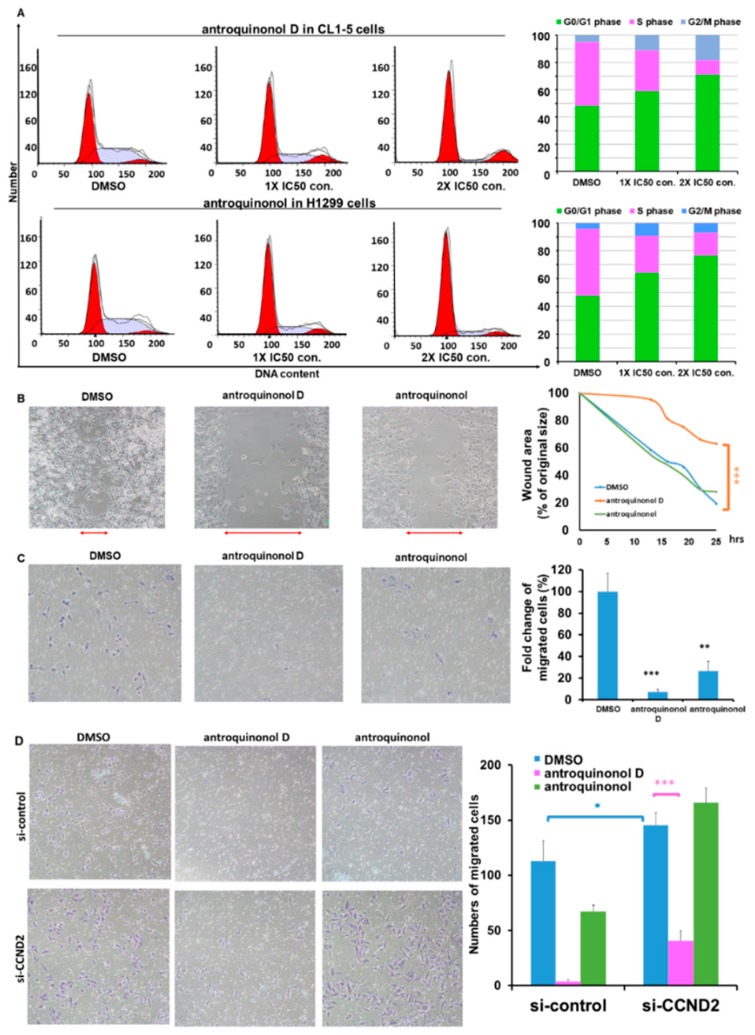
Antroquinonol D suppresses lung cancer cell migration mediated by low *CCND2* expression. (**A**) Cell cycle arrest was induced by antroquinonol D in CL1-5 lung cancer cells and by antroquinonol in H1299 lung cancer cells in a dose-dependent manner. Cancer cell migration was inhibited by antroquinonol D and antroquinonol assayed by the wound-healing assay (**B**) and Transwell assay (**C**). (**D**) The Transwell assay was used to analyze cancer cell migration ability following antroquinonol D and antroquinonol treatment for low *CCND2* expression. The data are presented as means ± SD * *p* ≤ 0.05, ** *p* ≤ 0.01, *** *p* ≤ 0.001.

To determine whether antroquinonol D induces cell cycle arrest, antroquinonol, and antroquinonol D were examined by flow cytometry combined with PI staining in CL1-5 and H1299 lung cancer cells. The data indicated that antroquinonol D induced G1 phase arrest in CL1-5 lung cancer cells in a dose-dependent manner (Figure 6A upper panel) and antroquinonol induced G1 phase arrest in H1299 lung cancer cells in a dose-dependent manner (Figure 6A lower panel).

### 2.7. Antroquinonol D Inhibits Cell Proliferation and Migration Ability through CCND2 Induction

Metastasis is the primary cause of mortality for most cancer patients. The motility of tumor cells is essential to the metastatic process. The highly metastatic human lung cancer cell line CL1-5 was used to examine cell motility while using wound-healing and Transwell assays. Wound-healing assays indicated that CL1-5 cells migrated significantly slower when treated with 15 μM antroquinonol D for three days than DMSO-treated control cells (Figure 6B). Transwell migration assays revealed that antroquinonol D and antroquinonol decreased the migration ability of CL1-5 cells by 92.46% and 73.6%, respectively (Figure 6C).

To determine whether antroquinonol D could repress CCND2 deficiency-mediated cancer cell migration, *CCND2* knockdown and Transwell assays were performed in H1299 lung cancer cells. The cell migration ability increased following *CCND2* knockdown (Figure 6B,D and Figure S6), and antroquinonol D was found to significantly suppress the migration ability following *CCND2* knockdown (Figure 6D).

## 3. Discussion

In this study, we identified a potential drug target, *CCND2*, for the treatment of TNBC and lung adenocarcinoma. Promoter hypermethylation of *CCND2* and low *CCND2* expression were associated with a poor prognosis, especially in NSCLC and breast cancer patients. Therefore, *CCND2* may serve as a potential drug target for the future of precision medicine. We also found that antroquinonol D, which is a demethylating agent and an analog of the fungal-derived Taiwanese natural product antroquinonol, could induce *CCND2* mRNA and protein expression, cell cycle arrest, and migration inhibition in lung and breast cancer cell lines.

The mechanism underlying the association of low *CCND2* expression with lung adenocarcinoma and TNBC is interesting. Among all genes, 33 aberrant gene expression levels were found in only lung adenocarcinoma and TNBC patients (i.e., not in other cancer patients). Using Ingenuity Pathway Analysis, we found that most genes were involved in carcinoma, adenocarcinoma, breast cancer, and TNBC (Figure 1). Mutations or amplifications of *CCND2* genes were found in patients with malignant gliomas or hematologic malignancies [31,32,33]. Thr280Ala-mutated CCND2 leads to the increased the phosphorylation of retinoblastoma protein, thereby causing significant cell cycle changes and increased proliferation of acute myeloid leukemia cell lines [31]. However, promoter hypermethylation of CCND2 is frequent in several solid cancers [15,30,34], such as breast cancer, lung cancer, and liver cancer (Figure 2). Reduced CCND2 expression promoted cell proliferation, and its overexpression inhibited prostate cancer cell growth [35]. *CCND2* knockdown in H1299 cells induced cell proliferation and metastasis (Figure 4A,B), suggesting that *CCND2* may act as a suppressor gene during solid tumor formation. Therefore, for future personalized medicine, a CCND2 inducer drug might be suitable for TNBC or female lung cancer and lung adenocarcinoma patients with low CCND2 expression, but not for patients with *CCND2* mutation or amplification.

Promoter hypermethylation of *CCND2* and low *CCND2* expression were also associated with a poor prognosis in overall lung and breast cancer, likely because most lung and breast cancer patients with a poor survival have lung adenocarcinoma and TNBC, respectively. Surprisingly, low *CCND2* expression in liver cancer was also associated with a poor prognosis. Thus, *CCND2* might also provide a new treatment target for inducer drugs for liver cancer patients.

Aberrantly methylated *CCND2* has been reported in breast, lung, gastric, and liver cancers [15,30,34,36]. Analysis of the Infinium Human Methylation 450K BeadChip assay data from TCGA revealed that *CCND2* promoter hypermethylation was more frequently aberrant in breast cancer, lung adenocarcinoma, and liver cancer than in adjacent normal tissues (Figure 2). However, breast cancer and lung adenocarcinoma showed a higher frequency of low *CCND2* mRNA expression than adjacent normal tissues. Low *CCND2* expression was infrequent in liver cancer (Figure 2), possibly because other mechanisms interfered with *CCND2* mRNA expression. Notably, almost no methylation was observed in several CpG sites of the *CCND2* promoter regions of adjacent normal tissues in all types of cancer, but hypermethylation was observed in breast cancer and lung adenocarcinoma. Additionally, *CCND2* hypermethylation was associated with a poor prognosis in breast and lung cancer patients. Multivariate Cox proportional hazard model analysis showed that *CCND2* expression was also significantly correlated with a poor survival (Table 2). High frequency (63.3%) of breast patients with methylated *CCND2* were also characterized by low *CCND2* mRNA expression (Table 1). Hypermethylation of *CCND2* could be successfully detected in the plasma of breast cancer patients compared with that in healthy persons. Therefore, aberrant circulating cell-free DNA methylation of *CCND2* may serve as a potential early diagnosis marker and poor prognosis marker in the future. However, DNA methylation can be changed when short-time exposure of tobacco carcinogen and other environmental factor [37,38]. Accordingly, the plasma cell-free circulating DNA samples will be isolated from blood cells and stored in −80 °C degree within 2 h. The weakness of this study is the cohort of 93 samples of Taiwanese breast cancer patients were analyzed for methylation of *CCND2* gene is not the same with the cohort of 18 patients were analyzed in patients’ plasma circulating cell free DNA. Samples collection design and process will be improved in the future study cohort. However, whether detecting the aberrant methylation of *CCND2* in the blood samples of patients can be applied as a noninvasive analytical method for early diagnosis, prognosis, or precision medicine in breast cancer and lung cancer patients is worth further massive screening and investigation.

*CCND2* is a potential target for lung and breast cancer in future personalized medicine. Aberrant methylation and other mechanisms that mediate *CCND2* low expression can be induced by antroquinonol D in breast cancer and lung cancer cells (Figure 5). Antroquinonol D has been reported to specifically inhibit DNMT1 activity [19]. Enzyme activity assays and molecular modeling have revealed that antroquinonol D is bound to the catalytic domain of DNMT1 and it competes for the same binding pocket in the DNMT1 enzyme as the cofactor SAM, but it does not compete for the binding pocket in the DNMT3B enzyme [19]. It is noteworthy that antroquinonol D still induced *CCND2* expression in lung cancer cell lines without aberrant *CCND2* promoter hypermethylation (Figure S4 and Figure 5G). Previous reports have shown that DNMT1 modulates gene expression without its catalytic activity through its interactions with histone-modifying enzymes [39]. Therefore, antroquinonol D induces *CCND2* through the inhibition of DNMT1-mediated transcriptional repression. Additionally, antroquinonol D may have other targets than DNMT1. It is vital to verify whether there are other targets of antroquinonol D through molecular modeling and compound-centric chemical proteomics [40].

However, the safety of antroquinonol D and its efficacy as an in vivo *CCND2* inducer needs further investigation. Antroquinonol is currently in phase II trials (USA and Taiwan) for the treatment of non-small cell lung carcinoma (NSCLC) and it was recently granted orphan drug status by the FDA for the treatment of pancreatic cancer and acute myeloid leukemia [26,27]. The pharmacological mechanism of antroquinonol treated in NSCLC patients is worthy to verify whether the treatment effect is mediated through CCND2 induction. Antroquinonol might be another choice as a candidate CCND2 inducer.

In conclusion, *CCND2* is a good diagnostic marker and drug target for breast and lung cancer. Antroquinonol D induces *CCND2* DNA demethylation, recovery of CCND2 expression, cancer cell death, and metastasis inhibition. Future studies should verify whether antroquinonol or antroquinonol D could be a potential drug for lung cancer or breast cancer patients with hypermethylation of *CCND2* or CCND2 low expression and improve the survival rate in these patients.

## 4. Materials and Methods

### 4.1. Patients and Plasma and Tissue Collection

For the CCND2 methylation assay in plasma, breast cancer patients who underwent diagnosis at Taipei Medical University were enrolled. Before clinical data and sample collection, written informed consent was obtained from all of the patients. Frozen human tissue samples were obtained from the Taipei Medical University (TMU) Joint Biobank. Sections of cancerous tissue and corresponding noncancerous tissues were reviewed by a senior pathologist. Clinical data on sex, personal and family medical history, tumor location, TNM tumor stage, tumor differentiation, and follow-up conditions, which were prospectively collected, were obtained from the TMU Joint Biobank.

### 4.2. Genomic DNA, Circulating Cell-Free DNA and RNA Extraction

Genomic DNA from the cell lines was prepared using the Blood & Tissue Genomic DNA Extraction Miniprep System (Viogene, Sunnyvale, CA, USA). Genomic DNA from matched pairs of primary tumors and adjacent breast tissues from the same patient was prepared using the QIAamp DNA Mini Kit (Qiagen, Bonn, Germany, Cat. No. 51306). The Circulating cell-free DNA was extracted from plasma. Briefly, 3.5 mL of plasma was isolated immediately from 10 mL of peripheral blood within 2 h. Circulating cell-free DNA (cfDNA) was extracted from plasma using the MagMAX Cell-Free DNA Isolation Kit, according to the manufacturer’s recommended protocol (Thermo Fisher Scientific, Austin, TX, USA) [41]. Total mRNA was extracted using the RNeasy Plus Mini Kit (Qiagen, Bonn, Germany, Cat. No. 74134). The DNA and RNA were quantified, and the purities were verified by measuring the A_260_ and A_260_/A_280_ ratios (which ranged from 1.8 to 2.0).

### 4.3. DNA Methylation Assays

Genomic DNA from matched pairs of primary tumors and adjacent breast tissues from the same patient and cfDNA from breast cancer patients were prepared using the QIAamp DNA Mini Kit (Qiagen, Bonn, Germany). The DNA methylation analyses in cell lines were determined using Illumina Methylation 450K array-based assays (Illumina, San Diego, CA, USA) and pyrosequencing. For the Illumina Methylation 450K array-based assays, the methylation scores were represented as ‘beta’ values that ranged from 0 (no methylation) to 1 (full methylation) by calculating the ratios of the methylated signal intensities to the sums of the methylated and unmethylated signal outputs. To analyze the methylation level of the *CCND2* gene, we designed primers targeting the promoter regions of *CCND2* and detected the DNA methylation levels through pyrosequencing. The primers were designed while using the MethPrimer [42] and Methyl Primer Express v1.0 (Thermo Fisher Scientific, Waltham, MA, USA) software programs. Polymerase chain reaction (PCR) was performed using biotinylated primers to convert the PCR products to single-stranded DNA templates. The pyrosequencing reactions and methylation quantification were performed using PyroMark Q24, which was provided by Mission Biotech Corporation, Taiwan. In plasma samples and tissues, probe-based methylation specific real-time PCR (qMSP) was used in DNA methylation analyses. After bisulfite conversion of genomic DNA using the EpiTect Fast DNA Bisulfite Kit (Qiagen, Bonn, Germany, Cat. No. 59826), according to the manufacturer’s recommended protocol, the DNA methylation level of *CCND2* was measured using qMSP and the LightCycler 480 system (Roche Applied Science, Penzberg, Germany). qMSP was performed using the SensiFAST™ Probe No-ROX Kit (Bioline, London, UK) and specific primers and the methyl-specific probe of *CCND2*. Normalized DNA methylation values, which were calibrated to the control group, were obtained using LightCycler Relative Quantification software (Version 2.0, Roche Applied Science).

The *ACTB* gene was used as a reference gene. *CCND2* was considered hypermethylated when the methylation level of *CCND2* relative to that of the *ACTB* gene was at least two-fold higher in breast tumors than in paired normal breast tissue samples. Patients with *CCND2* qMSP signals in plasma samples were considered *CCND2* hypermethylated patients. The specificity of *CCND2* methylation end products was confirmed by bisulfite sequencing (Figure S7). The primers are described in Table S6.

### 4.4. Real-Time RT–PCR

The mRNA expression levels were determined through real-time RT–PCR using a LightCycler 480 (Roche Applied Science, Mannheim, Germany). Real-time RT–PCR was performed using the LightCycler 480 Probe Master kit (Roche Applied Science) and specific *CCND2*, *GAPDH*, and other multiple TSG primers and the corresponding Universal Probe Library probe (Roche Applied Science). *GAPDH* was used as a reference gene. Normalized gene expression values, which were calibrated to the control group, were analyzed while using LightCycler Relative Quantification software (ver. 2.0, Roche Applied Science). The primers are described in Table S6.

### 4.5. The Cancer Genome Atlas Data Processing and Candidate Gene Selection

The results of the western cohort, RNA sequencing, Illumina Methylation 450K array-based assays, and clinical information, were based on data generated by The Cancer Genome Atlas (TCGA) Research Network (available online: http://cancergenome.nih.gov/). Gene expression was considered low when the RNA sequencing value of the tumors was at least 0.5 lower than that of the paired adjacent-normal tissues. A Venn diagram of aberrant gene expression profile in seven types of cancer was created and was visualized using InteractiVenn [43]. A comprehensive network and pathway analysis of candidate genes were identified by Ingenuity Pathway Analysis [44].

### 4.6. Statistical Analyses

All statistical analyses were performed using SPSS (SPSS Inc., Chicago, IL, USA). Pearson′s chi-squared test was used to compare lung and breast cancer patients in terms of *CCND2* methylation, RNA expression, and other clinical data, including age, gender, tumor type, TNM tumor stage, race, menopause state, and ER, PR, and HER2 status. *T*-test was also used to compare cells transfected with or without siCCND2 and those with or without drug treatment. The overall survival curves were calculated using the Kaplan–Meier method, and comparisons were performed using the log-rank and Wilcoxon test. The variables gender, race, age, tumor type, tumor stage, and menopause state were reported to be associated with prognosis in non-small cell lung cancer or breast cancer [45,46,47]. Multivariate Cox proportional hazards regression analyses (adjusted for age, gender, race, tumor subtype, tumor stage, and menopause) were further used to analyze the correlation between *CCND2* hypermethylation or CCND2 expression and survival in lung and breast cancer patients.

### 4.7. Antroquinonol and Antroquinonol D

The culture conditions of *Antrodia camphorata* and extraction method of antroquinonol and antroquinonol D were performed as previously reported [48]. From 500 g of the dried powder of cultured A. camphorata mycelium, 0.5 g of antroquinonol and 9.6 mg of antroquinonol D were obtained.

### 4.8. Cell. Lines and Drug Treatments

MDA-MB-231, CL1-5, A549, H1299, and IMR-90 cell lines were cultured in *DMEM* (Invitrogen, Carlsbad, CA, USA) supplemented with 10% fetal bovine serum (FBS) and 1% penicillin/streptomycin. The cells were treated with antroquinonol D, antroquinonol, or solvent control for the indicated times. Antroquinonol D and antroquinonol were dissolved in DMSO.

### 4.9. Sulphorhodamine B Assay

A sulphorhodamine B (SRB) assay was used to investigate the cell survival rate. The cells were seeded in 96-well plates at densities of 5000 cells/well and they were incubated with the indicated drugs for 72 h. The cells were fixed with 10% trichloroacetic acid, stained with SRB, and assessed by OD determination at 515 nm using a microplate reader. The data for antroquinonol D and antroquinonol were normalized to those for the solvent control (DMSO). The solvent controls were added in the same amounts as those that were used for the treatment drugs at the indicated concentrations.

### 4.10. Wound-Healing Assays for Migration Analysis

The wound-healing assays were performed using Culture Inserts (Ibidi, GmbH, Martinsried, Germany). Cell-free gaps of 500 μm remained after removing the Culture Inserts. After seeding the cells overnight, the Culture Inserts were detached, and images of the wounded areas were captured by an inverted microscope (Nikon, Surrey, UK) at 24 h.

### 4.11. Transwell Assays for Migration Analysis

The Transwell system comprises upper and lower chambers that are divided by a layer of membrane with 8-μm pores (Falcon, Colorado Springs, CO, USA). The 2 × 10^4^ CL1-5 cells were added to the upper chambers with 300 μL of serum-free medium with drugs, and the lower chambers were filled with 800 μL of DMEM medium with 10% FBS. After 16 h, the cells that did not invade were cleared using a scraper and they washed twice with PBS. Next, the cells were fixed and stained using 1% formaldehyde/PBS and crystal violet/ddH_2_O. A microscope (Nikon) captured five random images that were analyzed using ImageJ.

### 4.12. Cell. Cycle Distribution Assay

Cell cycle distribution was determined through flow cytometry. The CL1-5 and H1299 lung cancer cells (1 × 10^6^) were trypsinized and fixed with ethanol overnight at −20 °C. Next, the cells were washed and stained with a solution containing 20 μg/mL of propidium iodide, 200 μg/mL of RNase A, and 0.1% Triton X-100 for 30 min in the dark. The cell cycle distribution was investigated using a FACSCanto II flow cytometer (BD Biosciences, San Jose, CA, USA). The data were analyzed using ModFIT LT version 2.0 software (Verity Software House, Topsham, USA).

### 4.13. Cell. Lyses and Immunoblotting Analysis

For Western blotting assays, the cells were lysed on ice in radioimmunoprecipitation buffer (0.15 M NaCl, 0.05 M Tris-HCl [pH 7.4], 0.25% deoxycholic acid, 1 mM ethylenediaminetetraacetic acid, and 1% Igepal CA-630). The protein extracts were incubated with sodium dodecyl sulfate (SDS) gel-loading buffer (60 mmol/L of Tris base, 10% glycerol, 2% SDS). Samples containing equal amounts of protein (40 μg) were loaded onto a 10% SDS-polyacrylamide gel using electrophoresis and they were electroblotted onto Immobilon-P membranes (Millipore, Bedford, MA, USA) in transfer buffer. Immunoblotting was performed using antibodies against *CCND2* (1:250; Santa Cruz, CA, USA). β-Actin (1:5000; GeneTex, TX, USA) was used as an internal control.

## Figures and Tables

**Table 1:** The *CCND2* methylation level and mRNA expression, and its effects on prognosis in cancers from TCGA.

Characteristics	Breast Cancer		Lung Cancer			Liver Cancer	Esophageal Cancer	Gastric Cancer	Colon Cancer
	All ^1^	TNBC ^2^	All	AD ^3^	SCC ^4^				
Hypermethylation on *CCND2* gene (paired)	31/57 (54.4%)	3/6 (60.0%)	22/69 (31.9%)	14/29 (48.3%)	8/40 (20.0%)	26/50 (52.0%)	2/15 (13.3%)	1/2 (50.0%)	3/38 (7.9%)
Low *CCND2* mRNA expressions ^5^ (paired)	62/73 *** (84.9%)	7/7 *** (100%)	73/90 (81.1%)	45/49 *** (91.8%)	28/51 (54.9%)	21/50 (42.0%)	7/13 (53.9%)	22/32 (68.6%)	13/41 (31.7%)
% of methylation and low *CCND2*	43/49 (63.3%)	-	6/23 (26.1%)	5/16 (31.3%)	1/7 (14.3%)	6/41 (14.6%)	-	-	2/11 (18.2%)
Concordance ^6^ between methylation & mRNA expression	*p* = 0.002 **	-	*p* = 0.015 *	*p* = 0.029 *	*p* > 0.05	*p* > 0.05	-	-	*p* > 0.05
Prognosis ^7^	*p* = 0.048 *		*p* = 0.012 *			*p* = 0.011 *	*p* > 0.05	*p* > 0.05	*p* > 0.05

^1^ All subtypes of breast cancer. ^2^ triple-negative breast cancer. ^3^ Lung adenocarcinoma. ^4^ Lung squamous cell carcinoma. ^5^ These results were analyzed by the paired sample *T*-test. * *p* < 0.05; ** *p* < 0.01; *** *p* < 0. 001. ^6^ The concordance were analyzed by the Spearman′s rank correlation test. * *p* < 0.05; ** *p* < 0.01; *** *p* < 0. 001. ^7^ The survival rate were analyzed by the Kaplan Meier method and Log-Rank test. * *p* < 0.05; ** *p* < 0.01; *** *p* < 0. 001.

**Table 2:** Cox proportional hazard model of clinical parameters and CCND2 expression level associated with lung cancer and breast cancer.

**Lung ^2^**	**5-Year Overall Survival ^1^**					
**Cancer**	**Univariate Analysis**			**Multivariate Analysis**		
**Variable**	**HR**	**95% CI**	***p*-Value**	**HR**	**95% CI**	***p*-Value**
Gender	0.982	0.761–1.268	0.890	0.883	0.633–1.232	0.465
Race	1.088	0.767–1.543	0.635	1.158	0.742–1.805	0.519
Age	1.005	0.994–1.016	0.380	1.005	0.992–1.018	0.474
Tumor type	1.001	0.779–1.287	0.993	0.742	0.507–1.084	0.123
Stage ***	1.466	1.287–1.669	<0.001 ***	1.477	1.247–1.751	<0.001 ***
CCND2(RNA) ^3^	0.742	0.600–0.919	0.006 **	0.688	0.501–0.946	0.021 *
CCND2(methyl)	1.386	0.982–1.957	0.063	1.544	1.022–2.333	0.039 *
**Breast ^4^**	**10-Year Overall Survival ^1^**					
**Cancer**	**Univariate Analysis**			**Multivariate Analysis**		
**Variable**	**HR**	**95% CI**	***p*-Value**	**HR**	**95% CI**	***p*-Value**
Race	0.902	0.568–1.433	0.662	1.949	0.781–4.860	0.153
Age	2.055	1.366–3.091	0.001 ***	0.725	0.218–2.415	0.600
Tumor type	1.391	1.028–1.882	0.033 *	1.455	0.620–3.414	0.388
Stage	1.685	1.287–2.208	<0.001 ***	3.525	1.700–7.313	<0.001 ***
Menopause	1.256	0.904–1.746	0.174	1.757	0.904–3.415	0.096
CCND2(RNA) ^4^	0.594	0.353–1.000	0.050 *	0.173	0.064–0.467	0.001 ***
CCND2(methyl)	2.207	0.998–4.881	0.051	4.693	1.466–15.017	0.009 **

^1^ These results were analyzed by the Cox regression model. ^2^ The *CCND2* expression and methylation levels were derived from 758 lung cancer patients in TCGA data set. ^3^ CCND2(RNA) is *CCND2* mRNA expression; CCND2(methyl) is *CCND2* promoter DNA methylation levels. ^4^ The *CCND2* expression and methylation levels were derived from 436 breast cancer patients in TCGA data set. * *p* < 0.05; ** *p* < 0.01; *** *p* < 0.001.

## Supplementary Materials

The following are available online at http://www.mdpi.com/1422-0067/19/10/3096/s1.

## Abbreviations

antroquinonol D	3-demethoxyl antroquinonol
CCND2	cyclin D2
cfDNA	circulating cell-free DNA
HER2	growth factor receptor 2
NSCLC	non-small cell lung cancer
TCGA	The Cancer Genome Atlas
TMU	Taipei Medical University
TNBC	triple-negative breast cancer
