# Supplementary material for: Hypermethylation of CCND2 in Lung and Breast Cancer Is a Potential Biomarker and Drug Target
Source: Int J Mol Sci. 2018 Oct 10;19(10):3096. doi: 10.3390/ijms19103096 (PMC6213171; doi:10.3390/ijms19103096)
Supplement: Supplementary file 1 [file ijms-19-03096-s001.zip › ijms-346767-supplementary.pdf]

Table S1. The list of 15 genes with aberrant low expression in TNBC and lung adenocarcinoma, but not in other cancer, tumor tissues compared with adjacent normal tissues.

ID	Entrez Gene Name	Location	Type(s)
ABCC6	ATP binding cassette subfamily C member 6	Plasma Membrane	transporter
ANGPT4	angiopoietin 4	Extracellular Space	growth factor
CA3	carbonic anhydrase 3	Cytoplasm	enzyme
CACHD1	cache domain containing 1	Other	other
CCND2	cyclin D2	Nucleus	other
DOCK11	dedicator of cytokinesis 11	Cytoplasm	other
FAM47E	family with sequence similarity 47 member E	Cytoplasm	other
GPR109A	hydroxycarboxylic acid receptor 2	Plasma Membrane	G-protein coupled receptor
LIN7	Alin-7 homolog A, crumbs cell polarity complex component	Plasma Membrane	other
LPL	lipoprotein lipase	Cytoplasm	enzyme
PCDHGB7	protocadherin gamma subfamily B, 7	Other	other
PTGFR	prostaglandin F receptor	Plasma Membrane	G-protein coupled receptor
RAPGEF3	Rap guanine nucleotide exchange factor 3	Nucleus	other
TACC1	transforming acidic coiled-coil containing protein 1	Nucleus	other
TGFBR2	transforming growth factor beta receptor 2	Plasma Membrane	kinase

© 2000-2017 QIAGEN. All rights reserved. (Analyzed by Ingenuity Pathway Analysis)

Table S2. Demethylation in CCND2 mediated by antroquinonol D in MDA-MB-231 breast cancer cells.

Demethylation (△ Avg_Beta)[a]	Fold change of methylation[b] (%)	GENE NAME	Function	Reference
-0.200	33.343	CCND2	Cell cycle regulation	Highly methylation of *CCND2* in breast tumor than in normal tissue specimens (11).

a Demethylation (△ Avg_Beta) indicates that the change level of DNA methylation in specific gene between DMSO control and D-Antroquinonol treatment. The data was calculated by: D-Antroquinonol.Avg_Beta – DMSO.Avg_Beta. "Beta" scores are based on the ratio of methylated signal intensity to the sum of both methylated and unmethylated signals output.

b Fold change of DNA methylation was calculated by: [1-(D-Antroquinonol.Avg_Beta/DMSO.Avg_Beta)]*100.

Table S3. CCND2 mRNA expression in relation to the clinical parameters of lung cancer.[1.]

Characteristics	Total n	n	Low (%)	n	Normal n (%)	Total n	CCND2 methylation Low n(%)	CCND2 methylation High n(%)
			CCND2 mRNA					
**Overall**	1019	630	(61.8)	389	(38.2)	828	626(75.6)	202(24.4)
**Age**	1010	628	(62.2)	382	(37.8)	819	618(75.5)	201(24.5)
< 65	437	271	(62.0)	166	(38.0)	362	254(70.2)	108(29.8)$^{0.002}$
≥ 65	573	357	(62.3)	4	(37.7)	457	364(79.6)	93(20.4)
**Gender**	1019	630	(61.8)	389	(38.2)	828	626(75.6)	202(24.4)
Female	407	264	(64.9)	143	(35.1)	340	233(68.5)	107(31.5)$^{<0.001}$
Male	612	366	(59.8)	246	(40.2)	488	293(80.5)	95(19.5)
**Tumor type**	1019	630	(61.8)	389	(38.2)	828	626(75.6)	202(24.4)
Squamous carcinoma	504	250	(49.6)	254	(50.4)	370	358(96.8)	12(3.2)
Adenocarcinoma	515	380	(73.8)	135	(26.2)$^{<0.001}$	458	268(58.5)	190(41.5)$^{<0.001}$
**Tumor stage**	1007	621	(61.7)	386	(38.3)	820	622(75.9)	198(24.1)
I	520	308	(59.2)	212	(40.8)	420	320(76.2)	100(23.8)
II	285	178	(62.5)	107	(37.5)	247	188(76.1)	59(23.9)
III	169	108	(63.9)	61	(36.1)	129	96(74.4)	33(25.6)
IV	33	27	(81.8)	6	(18.2)	24	18(75.0)	6(25.0)
**Primary tumor**	1016	628	(61.8)	388	(38.2)	825	624(75.6)	201(24.4)
T1	284	167	(58.8)	117	(41.2)	245	192(78.4)	53(21.6)
T2	571	363	(63.6)	208	(36.4)	450	333(74.0)	117(26.0)
T3	118	76	(64.4)	42	(35.6)	101	76(75.2)	25(24.8)
T4	43	22	(51.2)	21	(48.8)	29	23(79.3)	6(20.7)
**Regional lymph nodes**	1013	626	(61.8)	387	(38.2)	823	623(75.7)	200(24.3)
N0	663	399	(60.2)	264	(39.8)	546	419(76.7)	127(23.3)
N1	229	136	(59.4)	93	(40.6)	182	135(74.2)	47(25.8)
N2	114	86	(75.4)	28	(24.6)	94	68(72.3)	26(27.7)
N3	7	5	(71.4)	2	(28.6)$^{0.014}$	1	1(100.0)	0(0.0)
**Distant metastasis**	1016	629	(61.9)	387	(38.1)	825	625(75.8)	200(24.2)
No distant metastasis	983	602	(61.2)	381	(38.8)	801	607(75.8)	18(24.2)
Distant metastasis	33	27	(81.8)	6	(18.2)$^{0.017}$	24	18(75.0)	6(25.0)

1. These results were analyzed by the Pearson X^2 test. *P* values with significance are shown as superscripts.
2. When the CCND2 expression level in lung tumors was less than half of the mean of CCND2 expression levels in adjacent normal lung tissues was defined as low expression from TCGA data set using RNA sequencing analysis.
3. *, $P < 0.05$; **, $P < 0.001$.

Table S4. CCND2 mRNA expression and promoter hypermethylation in relation to the clinical parameters of breast cancer.[1.]

Characteristics	Total	CCND2 mRNA					CCND2 Methylation			
		Low		High			Low		High	
	n	n	(%)		n (%)			n (%)		n (%)
**Overall**	761	183	(24.0)	578	(76.0)	623	86	(13.8)	537	(86.2)
**Age**	761	183	(24.0)	578	(76.0)	623	537	(86.2)	86	(13.8)
< 65	551	**121**	**(22.0)**	**430**	**(78.0)**$^{0.029}$	453	**399**	**(88.1)**	**54**	**(11.9)**$^{0.026}$
≥ 65	210	**62**	**(29.5)**	**148**	**(70.5)**	170	**138**	**(81.2)**	**32**	**(18.8)**
**Race**	525	117	(22.3)	407	(77.7)	433	371	(85.7)	62	(14.3)
White	421	**82**	**(19.5)**	**33.9**	**(80.5)**$^{0.003}$	338	**292**	**(86.4)**	**46**	**(13.6)**$^{0.722}$
Black/African American	72	**27**	**(37.5)**	**45**	**(62.5)**	64	**53**	**(82.8)**	**11**	**(17.2)**
Asian	32	**8**	**(25.0)**	**24**	**(75.0)**	31	**26**	**(83.9)**	**5**	**(16.1)**
**Menopause State**	480	117	(22.3)	407	(77.7)	404	347	(85.9)	57	(14.1)
Premenopause	123	**23**	**(18.7)**	**100**	**(81.3)**$^{0.536}$	103	**90**	**(87.4)**	**46**	**(13.6)**$^{0.242}$
Perimenopause	16	**3**	**(18.8)**	**13**	**(81.3)**	14	**14**	**(100.0)**	**0**	**(0.0)**
Postmenopause	341	**79**	**(23.2)**	**262**	**(76.8)**	287	**243**	**(84.7)**	**11**	**(15.3)**
**Histological Type**	714	166	(23.2)	548	(76.8)	582	347	(85.9)	57	(14.1)
ILC	170	**25**	**(14.7)**	**145**	**(85.3)**$^{<0.001}$	152	**125**	**(82.2)**	**27**	**(17.8)**$^{0.356}$
IDC	504	**128**	**(25.4)**	**376**	**(74.6)**	400	**347**	**(86.8)**	**53**	**(13.3)**
Mucinous Carcinoma	15	**11**	**(73.3)**	**4**	**(26.7)**	14	**11**	**(78.6)**	**3**	**(21.4)**
Mixed type	25	**2**	**(8.0)**	**23**	**(92.0)**	16	**15**	**(93.8)**	**1**	**(6.3)**
**Tumor Stage**	535	117	(21.9)	418	(78.1)	440	379	(86.1)	61	(13.9)
I	86	**14**	**(16.3)**	**72**	**(83.7)**$^{0.371}$	70	**62**	**(88.6)**	**8**	**(11.4)**$^{0.181}$
II	299	**73**	**(24.4)**	**226**	**(75.6)**	241	**200**	**(83.0)**	**41**	**(17.0)**
III	142	**28**	**(19.7)**	**114**	**(80.3)**	123	**112**	**(91.1)**	**11**	**(8.9)**
IV	8	**2**	**(25.0)**	**6**	**(75.0)**	6	**5**	**(83.3)**	**1**	**(16.7)**
**ER**	535	121	(22.6)	414	(77.4)	443	381	(86.0)	62	(14.0)
Negative	117	**33**	**(28.2)**	**84**	**(71.8)**$^{0.102}$	103	**92**	**(89.3)**	**11**	**(10.7)**$^{0.268}$
Positive	418	**88**	**(21.1)**	**330**	**(78.9)**	340	**289**	**(85.0)**	**51**	**(15.0)**
**PR**	533	121	(22.7)	412	(77.3)	442	380	(86.0)	62	(14.0)
Negative	169	**50**	**(29.6)**	**119**	**(70.4)**$^{0.010}$	143	**123**	**(86.0)**	**11**	**(14.0)**$^{0.986}$
Positive	364	**71**	**(19.5)**	**293**	**(80.5)**	299	**257**	**(86.0)**	**51**	**(14.0)**
**HER2**	463	99	(21.4)	364	(78.6)	385	331	(86.0)	54	(14.0)
Negative	404	**91**	**(22.5)**	**313**	**(77.5)**$^{0.117}$	340	**297**	**(87.4)**	**43**	**(12.6)**$^{0.032}$
Positive	59	**8**	**(13.6)**	**51**	**(86.4)**	45	**34**	**(75.6)**	**11**	**(24.4)**
**TRBC**	515	115	(22.3)	400	(77.7)	426	365	(85.7)	61	(14.3)
Negative	76	**25**	**(32.9)**	**51**	**(67.1)**$^{0.017}$	68	**59**	**(86.8)**	**9**	**(13.2)**$^{0.781}$
Positive	439	**90**	**(20.5)**	**349**	**(79.5)**	358	**306**	**(85.5)**	**52**	**(14.5)**

1. These results were analyzed by the Pearson X^2 test. *P* values with significance are shown as superscripts.
2. When the CCND2 expression level in breast tumors was less than half of the mean of CCND2 expression levels in adjacent normalbreast tissues was defined as low expression from TCGA data set using RNA sequencing analysis.

Table S5. Methylation levels of CCND2 promoter through pyrosequencing.

Cells	Treatment	Position	1	2	3	4	5	6
CL1-5	DMSO	Methylation (%)	7	10	5	17	11	10
	Antroquinonol	Methylation (%)	10	11	6	16	14	10
	Antroquinonol D	Methylation (%)	11	12	5	17	13	10
H1299	DMSO	Methylation (%)	16	13	7	23	25	27
	Antroquinonol	Methylation (%)	14	12	7	20	24	24
	Antroquinonol D	Methylation (%)	14	26	8	22	26	25

Table S6. List of primer sequences and reaction conditions used in the study.

**Real-time RT-PCR[a]**		**Sequence (5' to 3')**	**Probe**	**Tm (ºC)**
CCND2	Forward	GGACATCCAACCCTACATGC	#49	60
	Reverse	CGCACTTCTGTTCCTCACAG		
COL4A2	Forward	CCAGGACAGAAAGGAGACCA	#1	60
	Reverse	GGTGTGATGCCTGGGAAC		
FOXN3	Forward	ATCAGGCACCTTCTCCTCAA	#15	60
	Reverse	TCTCAGCAGTGTGAGCCATC		
GAPDH	Forward	AGCCACATCGCTCAGACAC	#60	60
	Reverse	GCCCAATACGACCAAATCC		
RPS6KA2	Forward	CAGTTGCTGGAACTATGGAAAA	#39	60
	Reverse	TCCAGGTTGAGATCCTCCTCT		
RXRA	Forward	ACATGCAGATGGACAAGACG	#26	60
	Reverse	TCGAGAGCCCCTTGGAGT		
TAOK1	Forward	TCGTAATCGAGAACACTTTGCT	#56	60
	Reverse	CTTGCATTTGCCTCGTAACC		
FANCC	Forward	ATCAGGCACCTTCTCCTCAA	#15	60
	Reverse	TCTCAGCAGTGTGAGCCATC		
CACNA1A	Forward	TCGCCGAGGATGAAACTG	#32	60
	Reverse	GCTTTTCTTTATGGTGGTTCTCC		
CDH15	Forward	GGACATCGCCGACTTCAT	#75	60
	Reverse	GGCACACTGGGGTCACTATC		
ASB9	Forward	AAACAAGGGGGCATGGAT	#72	60
	Reverse	ACCAATCAGACACAGCATCG		
DAPK1	Forward	CCCAGTTGAAGAACCCATAGC	#49	60
	Reverse	CGAGGAACATTCATGATGTCAG		
FOXN3	Forward	GCTTTTGAATACTCCCCCTGA	#65	60
	Reverse	GCGCTCCATTTTGGATCA		
FOXP3	Forward	GAGAAGCTGAGTGCCATGC	#20	60
	Reverse	AGCCCTTGTCGGATGATG		
HOXD10	Forward	CTGAGGTCTCCGTGTCCAGT	#2	60
	Reverse	GCTGGTTGGTGTATCAGACTTG		
LRP1B	Forward	CGTGACTTGTGTCTCCCAGA	#76	60
	Reverse	GGGGCACTTGATTTCTACCTC		
RARB	Forward	TTGTGTTCACCTTTGCCAAC	#51	60
	Reverse	TTTGTCGGTTCCTCAAGGTC		
RHOBTB3	Forward	CCACCTCAACTTGAACAACCA	#41	60
	Reverse	GGCAGCAGAACAGCAAGTTA		
SOX4	Forward	AGCCGGAGGAGGAGATGT	#20	60
	Reverse	TTCTCGGGTCATTTCCTAGC		
TBC1D8	Forward	CCGAGCCAGATCACCAAG	#85	60
	Reverse	GAAAGCCCGGAAGAACTCA		
TCERG1L	Forward	ACATGCTGCTGGAGAGAGG	#14	60
	Reverse	GGTCAAACACGATTTTGTGTAATTC		
THBS4	Forward	CCTGAGACCATTGAATTGAGG	#6	60
	Reverse	ACCAGCTTCAGCTCTTCCAA		
		Pyrosequencing		
CCND2	Bis-Forward	GAGGGAGGGAGAGATTGAAAG		60.2
	Bio- Reverse	ATCCTCCCCTTAAAACTAATCT		58.5
	Seq- Forward	GGGAGGAGGAATTAGA		44.7
		qMSP		
CCND2	Forward	CGTTAGAGTACGTGTTAGGGTCGATC		60
	Reverse	CGAAAACATAAAACCTCCACGCT		60
	LNA-probe	56-FAM/AA TCG CCG C/ZEN/C AAC ACG /3IABKFQ		70

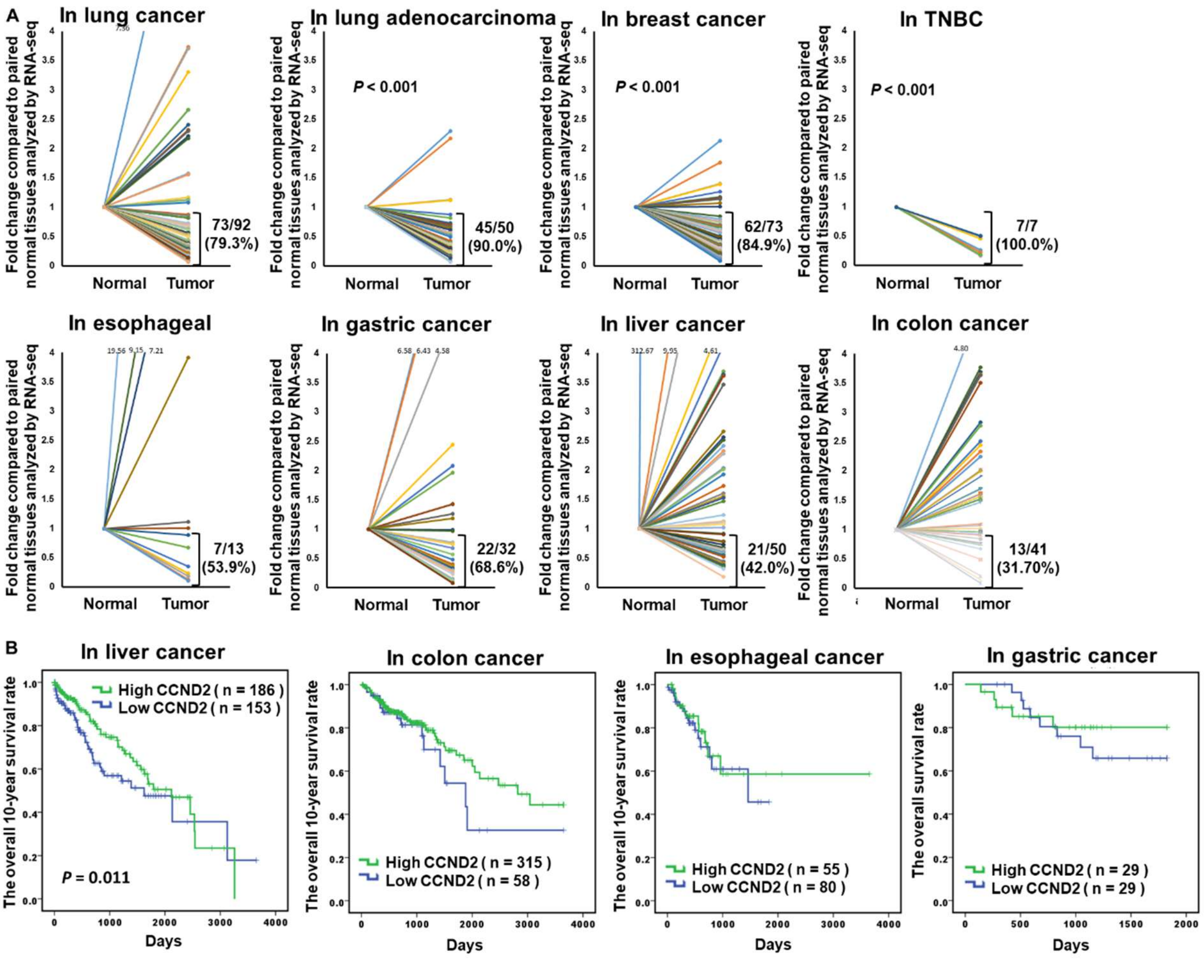

Figure S1 *CCND2* mRNA expression level and its correlation with survival in lung, breast, liver, colon, and gastric cancers. Data for mRNA expression level assayed through RNA sequencing and survival data were collected from TCGA. (**A**) The fold changes of normal tissues and tumors for RNA sequencing were normalised using the data of paired adjacent normal tissues. (**B**) Kaplan–Meier survival curves were used to compare the overall and 10-year survival between cancer patients with low and high *CCND2* mRNA expression level in lung cancer, breast cancer, liver cancer, and colon cancer. Because the follow-up time for gastric cancer patients was less than 10 years, only overall survival was analysed in these patients.

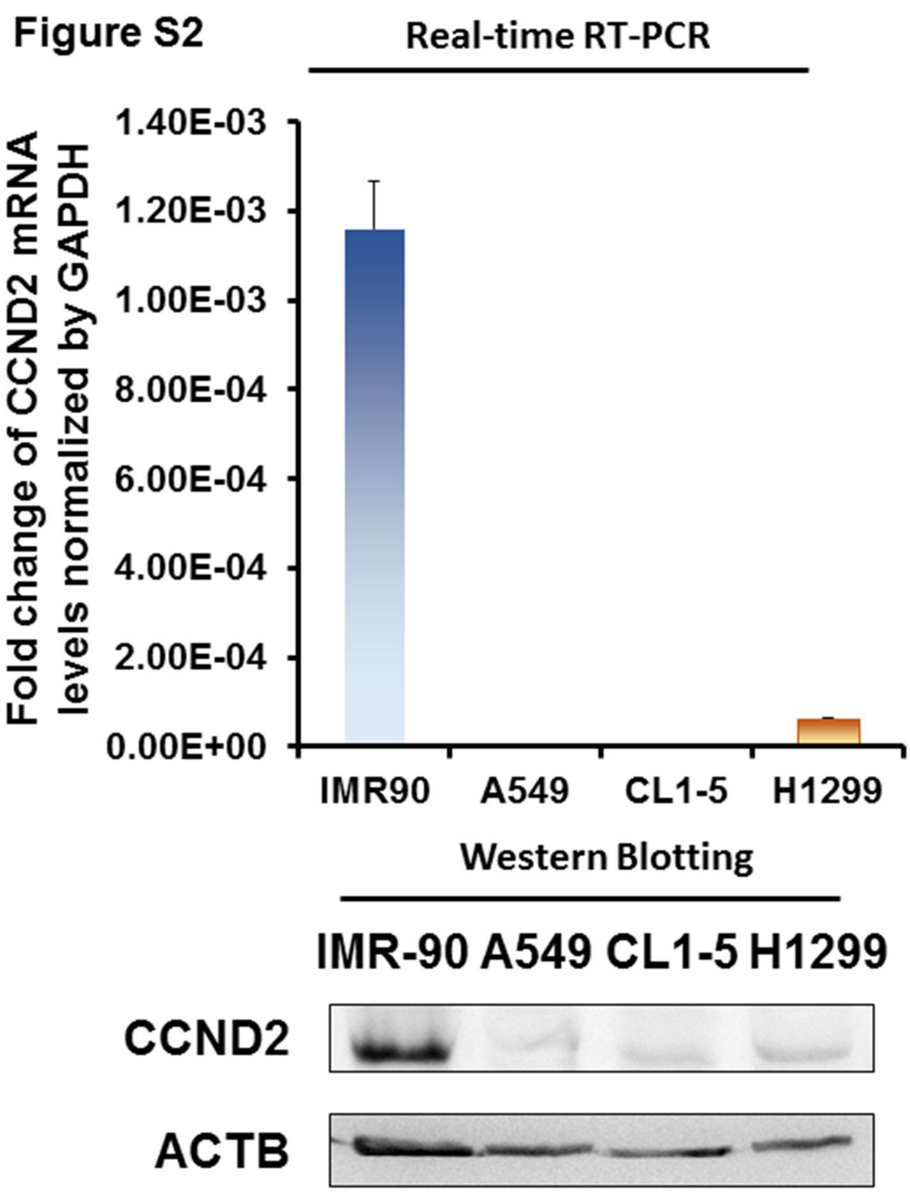

Figure S2. *CCND2* mRNA and protein expression in lung cancer and normal cell lines.

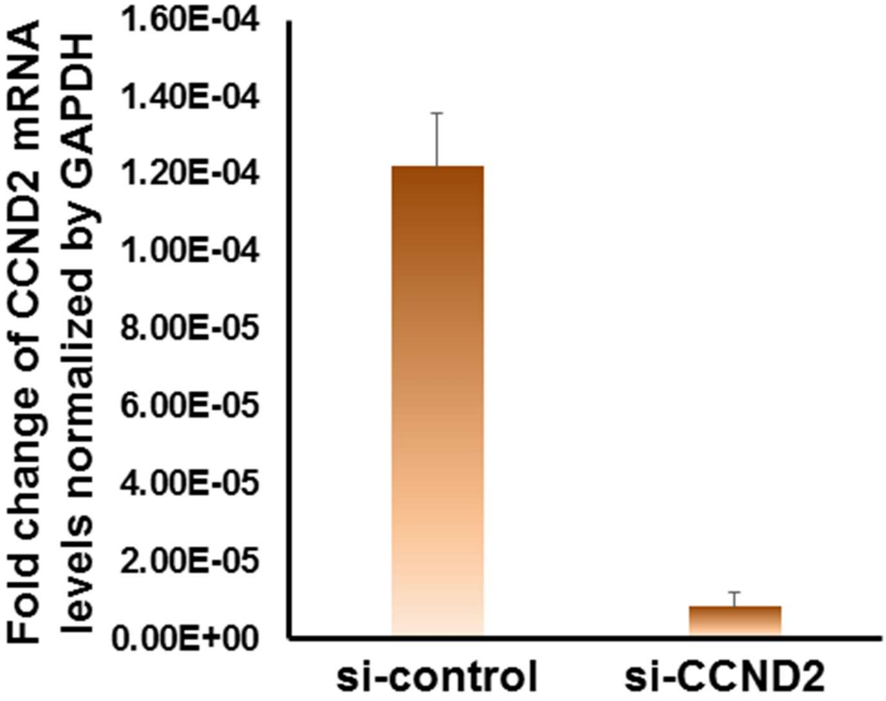

Figure S3. *CCND2* mRNA expression level when treated with si-control or si-*CCND2*.

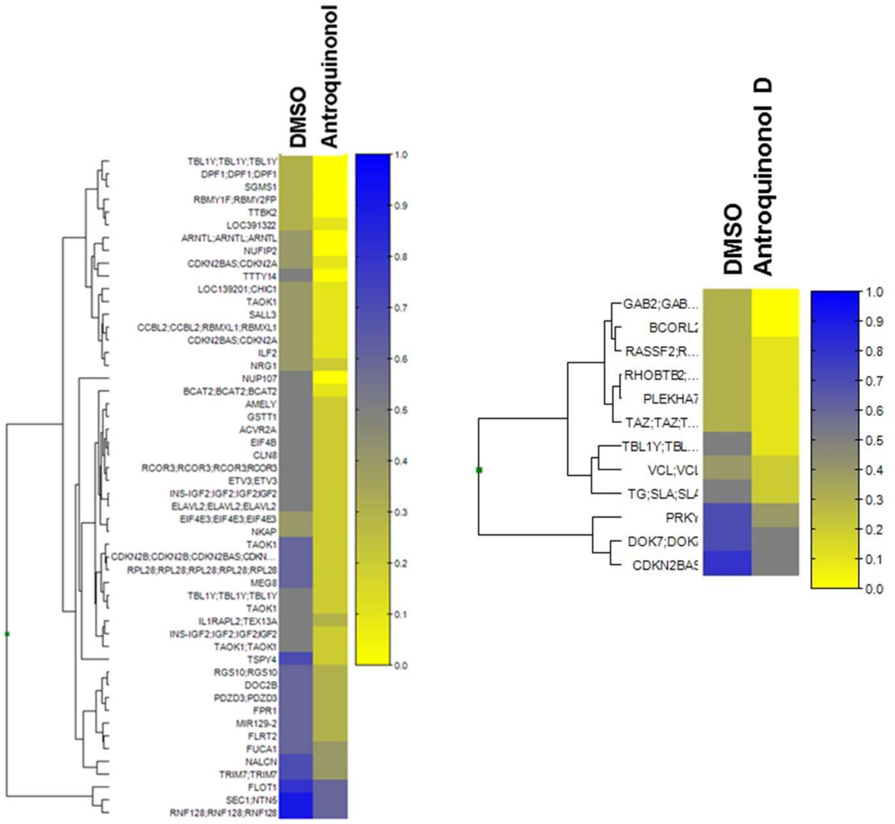

Figure S4. Heatmap of demethylation genes followed by antroquinonol D and antroquinonol.

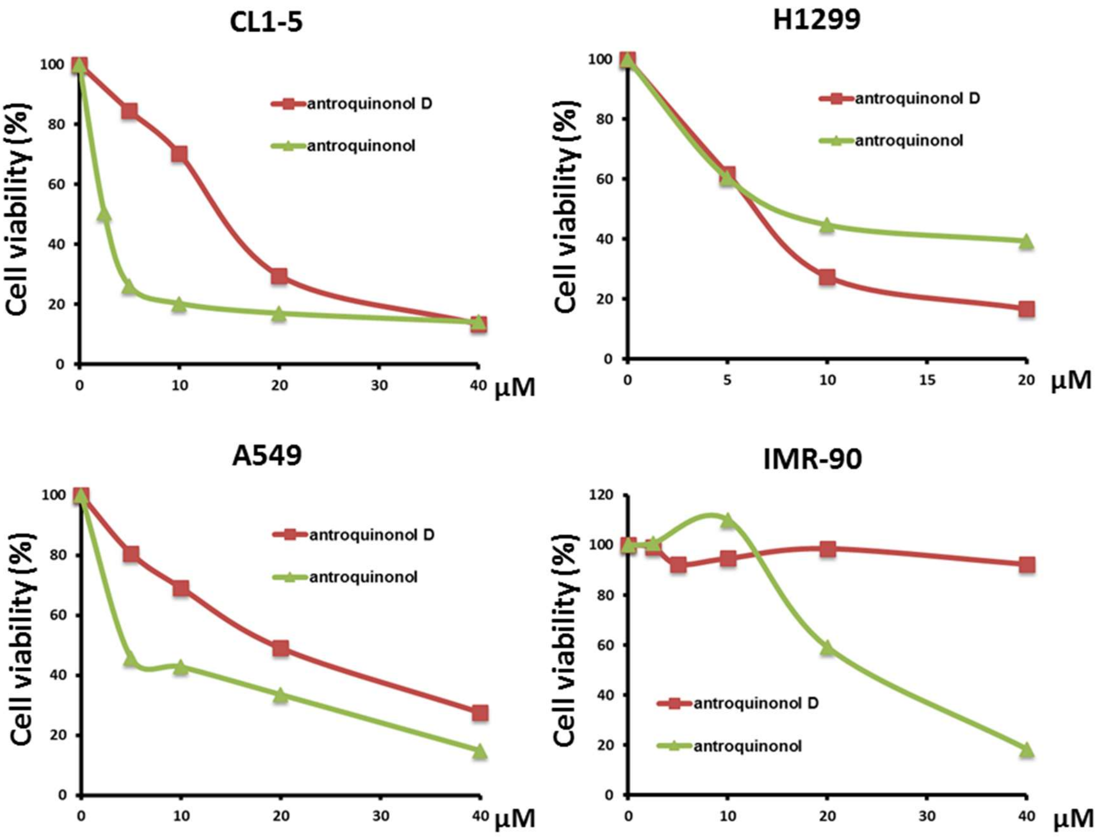

Figure S5. Cell viability curve of antroquinonol D and antroquinonol.

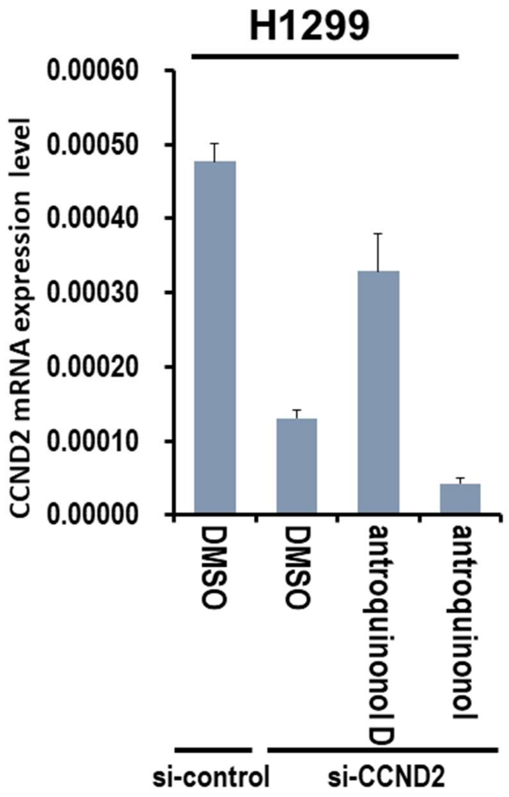

Figure S6. The CCND2 mRNA expression followed by si-CCND2 and drug treatment.

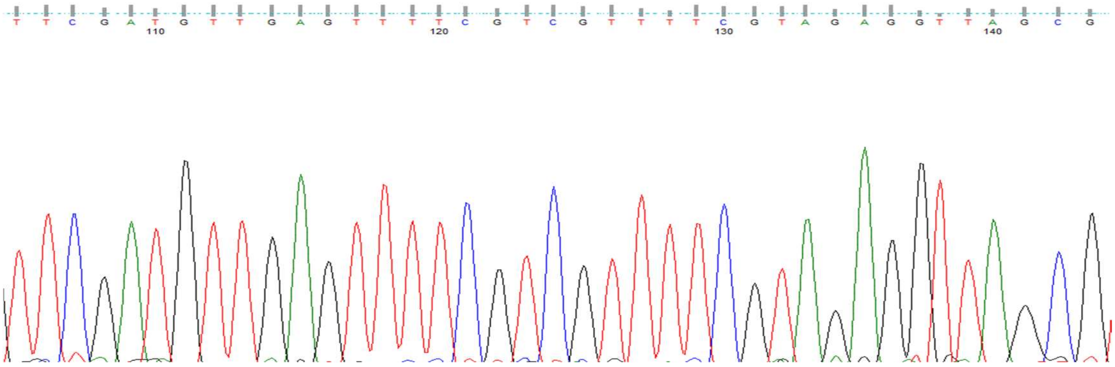

Figure S7. The qPCR end product for *CCND2* methylation was analyzed by bisulfite sequencing.

References

1. Ganzinelli M, Mariani P, Cattaneo D, et al. Expression of DNA repair genes in ovarian cancer samples: biological and clinical considerations. Eur J Cancer 2011; 47: 1086-94.
2. Morey Kinney SR, Smiraglia DJ, James SR, Moser MT, Foster BA, Karpf AR. Stage-specific alterations of DNA methyltransferase expression, DNA hypermethylation, and DNA hypomethylation during prostate cancer progression in the transgenic adenocarcinoma of mouse prostate model. Mol Cancer Res 2008; 6: 1365-74.
3. Castro M, Grau L, Puerta P, et al. Multiplexed methylation profiles of tumor suppressor genes and clinical outcome in lung cancer. J Transl Med 2010; 8: 86.
4. Yamada S, Nomoto S, Fujii T, et al. Frequent promoter methylation of M-cadherin in hepatocellular carcinoma is associated with poor prognosis. Anticancer Res 2007; 27: 2269-74.
5. Tokuoka M, Miyoshi N, Hitora T, et al. Clinical significance of ASB9 in human colorectal cancer. Int J Oncol 2010; 37: 1105-11.
6. Bignone PA, Lee KY, Liu Y, et al. RPS6KA2, a putative tumour suppressor gene at 6q27 in sporadic epithelial ovarian cancer. Oncogene 2007; 26: 683-700.
7. He XP, Li ZS, Zhu RM, et al. Effects of recombinant human canstatin protein in the treatment of pancreatic cancer. World J Gastroenterol 2006; 12: 6652-7.
8. L'Esperance S, Popa I, Bachvarova M, et al. Gene expression profiling of paired ovarian tumors obtained prior to and following adjuvant chemotherapy: molecular signatures of chemoresistant tumors. Int J Oncol 2006; 29: 5-24.
9. Chen YJ, Liao CT, Chen PJ, et al. Downregulation of Ches1 and other novel genes in oral cancer cells chronically exposed to areca nut extract. Head Neck 2011; 33: 257-66.
10. Chang H, Mohabir N, Done S, Hamel PA. Loss of ALX4 expression in epithelial cells and adjacent stromal cells in breast cancer. J Clin Pathol 2009; 62: 908-14.
11. Zhu W, Qin W, Hewett JE, Sauter ER. Quantitative evaluation of DNA hypermethylation in malignant and benign breast tissue and fluids. Int J Cancer 2010; 126: 474-82.
